# A genome-wide association study links small-vessel ischemic stroke to autophagy

**DOI:** 10.1038/s41598-017-14355-3

**Published:** 2017-11-09

**Authors:** Tsong-Hai Lee, Tai-Ming Ko, Chien-Hsiun Chen, Yeu-Jhy Chang, Liang-Suei Lu, Chien-Hung Chang, Kuo-Lun Huang, Ting-Yu Chang, Jiann-Der Lee, Ku-Chou Chang, Jen-Tsung Yang, Ming-Shien Wen, Chao-Yung Wang, Ying-Ting Chen, Tsai-Chuan Chen, Shu-Yu Chou, Ming-Ta Michael Lee, Yuan-Tsong Chen, Jer-Yuarn Wu

**Affiliations:** 1 Chang Gung Memorial Hospital, Linkou Medical Center, and Chang Gung University College of Medicine, Taoyuan, Taiwan; 2 Institute of Biomedical Sciences, Academia Sinica, Taipei, Taiwan; 3 Graduate Institute of Integrated Medicine, College of Chinese Medicine, China Medical University, Taichung, Taiwan; 4 School of Chinese Medicine, China Medical University, Taichung, Taiwan; 5 Chang Gung Memorial Hospital, Chiayi Branch, and Chang Gung University College of Medicine, Chiayi, Taiwan; 6 Chang Gung Memorial Hospital, Kaohsiung Medical Center, and Chang Gung University College of Medicine, Kaohsiung, Taiwan; 7 Genomic Medicine Institute, Geisinger Health System, Danville, Pennsylvania USA; 8 Department of Pediatrics, Duke University Medical Center, Durham, North Carolina USA

## Abstract

Genome-wide association studies (GWAS) can serve as strong evidence in correlating biological pathways with human diseases. Although ischemic stroke has been found to be associated with many biological pathways, the genetic mechanism of ischemic stroke is still unclear. Here, we performed GWAS for a major subtype of stroke—small-vessel occlusion (SVO)—to identify potential genetic factors contributing to ischemic stroke. GWAS were conducted on 342 individuals with SVO stroke and 1,731 controls from a Han Chinese population residing in Taiwan. The study was replicated in an independent Han Chinese population comprising an additional 188 SVO stroke cases and 1,265 controls. Three SNPs (rs2594966, rs2594973, rs4684776) clustered at 3p25.3 in *ATG7* (encoding Autophagy Related 7), with *P* values between 2.52 × 10^−6^ and 3.59 × 10^−6^, were identified. Imputation analysis also supported the association between *ATG7* and SVO stroke. To our knowledge, this is the first GWAS to link stroke and autophagy. *ATG7*, which has been implicated in autophagy, could provide novel insights into the genetic basis of ischemic stroke.

## Introduction

Stroke is known to be the second leading cause of death and a major cause of disability worldwide^1^. Although traditional vascular risk factors such as hypertension, diabetes, atrial fibrillation, and cigarette smoking are common in stroke, stroke incidence^2^ and subtype distribution^3^ are different among ethnicities. It is possible that a non-traditional risk factor such as genetic predisposition might be important. Data from twin and family history studies have suggested a role for genetic factors in stroke risk^4,5^. A previous study of vascular disease reported that family history is an independent risk factor for SVO, especially in cases presenting before the age of 65, suggesting the involvement of underlying genetic components in the development of SVO^6^. A GWAS conducted using a Japanese cohort with ischemic stroke identified a genetic variant in *PRKCH* ^7^; however, a meta-analysis of GWAS data from a Caucasian population found no association between ischemic stroke and *PRKCH* genetic variants^8^.

Compared with recent advances in high throughput genotyping for other subtypes of ischemic stroke^9–11^, gene discovery for SVO has progressed slowly because of etiologic heterogeneity and variations among different ethnic backgrounds. By exploiting these phenotypically more homogeneous classifications, a GWAS conducted using a single population may identify a genetic association specific to SVO. Thus, in the present study, we aimed to identify genetic correlations for SVO using a GWAS within a Han Chinese population.

## Methods

### Ethical statement

All methods were performed in accordance with the relevant guidelines and regulations. The study was approved by the Institutional Review Board and the Ethics Committee of the Institutional Review Board of Chang Gung Healthcare System and Academia Sinica, Taiwan. Written informed consents were obtained from the subjects or their family members in accordance with institutional requirements and Declaration of Helsinki principles.

### Study subjects and phenotype definitions

Individuals with SVO stroke (n = 530) (comprising 342 SVO stroke cases from the GWAS and 188 SVO stroke cases from the replication study) were recruited from the three branch hospitals of Chang Gung Healthcare System, Linkou, Chiayi and Kaohsiung, in collaboration with the Translational Resource Center (TRC) for Genomic Medicine of Taiwan. These three branch hospitals cover a population of six million in Taiwan with a total of 3,300 annual ischemic stroke patients. SVO was defined by the presence of subcortical, hypodense lesions with a diameter of <15 mm with accompanying clinical lacunar syndrome. The medical information and blood samples of all cases were centralized in Linkou CGMH, and the SVO stroke subtype was classified according to modified TOAST criteria^12^ by single physician, TH Lee, to prevent from interobserver discrepancy. Besides the criteria of clinical presentations and lacunas in brain images, only cases with diameter stenosis <30% in extracranial carotid artery confirmed by carotid ultrasound and/or in intracranial carotid artery by angiography (magnetic resonance, computed tomography or digital subtraction) were included for analysis. The control subjects (1,731 in the discovery study and 1,265 in the replication study) from the GWAS were randomly selected from the Taiwan Han Chinese Cell and Genome Bank in Taiwan. These controls were presumably disease-free as reported previously^13^.

### Genotyping and quality control

Genomic DNA was extracted from blood using a Puregene DNA Isolation Kit (Gentra Systems). Each individual was genotyped using the Axiom Genome-Wide CHB (with a total of 642,832 SNPs) according to manufacturer’s protocols by the National Center for Genome Medicine (NCGM) at Academia Sinica. All sample call rates were >98.69%, and the mean individual sample call rate was 99.5 ± 0.26%. First-degree relatives (parent-offspring and full sibling pairs) in SVO stroke cases and in control samples were identified by kinship analysis and were excluded from further analysis. Genotyping quality control for each SNP was further determined by the total call rate (successful call rate) and MAF in SVO stroke cases and controls. SNPs were excluded from further analysis if only one allele appeared in SVO stroke cases and controls, if the total call rate was <0.95, or if the total MAF was <0.05 and the total call rate was <0.99.

### Statistical analysis

**Figure 1:**
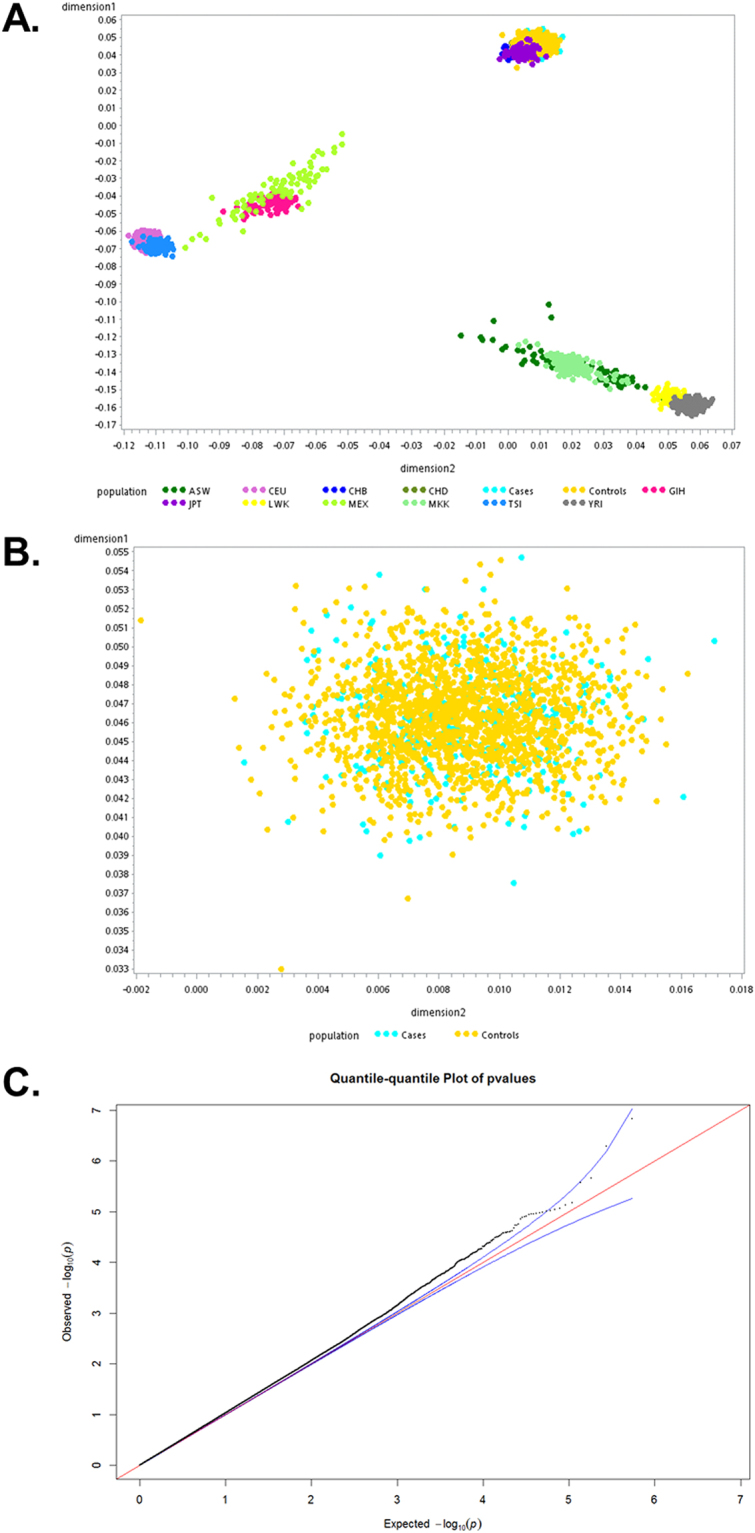
Multidimensional scaling analysis. **(A**) Results of the multidimensional scaling analysis of the GWAS samples with HapMap populations. (**B**) Results of the multidimensional scaling analysis of the GWAS samples with the GWAS samples only. (**C**) Q-Q plot of the *P* values in a Cochran-Armitage trend test. Lambda value is 1.09.

The statistical method used for GWAS analysis has been well-established in our previous study^9,14^. Detection of possible population stratification that could influence association analysis was carried out using EIGENSTRAT 2.0. We estimated the variance inflation factor for genomic controls. Genome-wide association analysis and GC correction were carried out to compare allele and genotype frequencies between cases and controls using the Cochran-Armitage trend test. A quantile-quantile (Q-Q) plot was used to determine *P* value distribution (Fig. 1). The adjustment for principle components suggested that inflation was not due to population stratification.

### GWAS validation and replication

The top three SNPs (*P* < 1 × 10^−4^) from the genome-wide association analysis of the 342 SVO stroke cases and controls were further validated using MALDI-TOF mass spectrometry (MassARRAY, Sequenom) (Supplementary Table 1). In addition, we also validated other 30 SNPs (*P* < 1 × 10^−2^) in the discovery stage (Supplementary Table 1). SNP genotypes with over 98% success rate and over 98% concordance between the two platforms were then genotyped. An additional 188 SVO stroke cases were used for the replication study.

### Imputation

For enhancement of the coverage, untyped SNPs were imputed by IMPUTE2 using 1000 Genomes reference panel^15–17^. In the pre-phasing step, we set up the haplotypes inferences via SHAPEIT method for optimizing the imputation procedure^18^. For elimination of edge effects, we expanded 500 kb buffer region on each side of imputation region. We determined the uncertainty of imputed genotypes based on likelihood scoring in SNPTEST v2 and frequentist association test of the additive model. We further validated the top imputed SNP by direct genotyping.

### Data availability

The datasets generated during and/or analysed during the current study are available in the International Stroke Genetics Consortium repository, http://www.cerebrovascularportal.org/. The name of the dataset is “SVO-Han-population Taiwan-NCGM”.

## Results

### Study populations

Characteristics of the SVO stroke groups are shown in Table 1. The mean age of the SVO group in the GWAS (discovery) group and replication group was 57.9 years old and 56.0 years old, respectively. The ratio of males to females in the GWAS group and replication groups was 67.3% and 67.6%, respectively. For other stroke risk factors, there was no significant difference between the GWAS group and the replication group.

**Table 1:** Baseline Demographic Summary of Patients

	n		Discovery (n = 342)	n		Replication (n = 188)
Median Age (IQR)	342	57.9	(48.3 − 67.0)	183	56	(44.0 − 67.0)
Sex-Male (%)	342	67.30%		188	67.60%	
Hypertension (%)	340	77.60%		187	78.10%	
Diabetes mellitus (%)	340	29.10%		187	32.60%	
Alcohol (%)	329	22.20%		173	15.00%	
Family history of stroke (%)	327	41.90%		166	42.80%	
Median HDL-C (IQR)	331	44	(36.0 − 53.0)	175	42	(35.0 − 49.0)
Median LDL-C (IQR)	328	110	(93.0 − 135.0)	174	115	(91.0 − 137.0)
Median VLDL (IQR)	133	26	(19.0 − 42.0)	91	30	(20.0 − 37.3)
Median Triglyceride (IQR)	341	131	(99.0 − 188.0)	188	145	(105.0 − 185.5)
Median Cholesterol (IQR)	341	185	(164.0 − 212.0)	188	187.5	(162.8 − 216.3)
Median Uric acid (IQR)	329	5.9	(4.9 − 7.1)	177	6.6	(5.1 − 7.6)

### Assessment of population stratification

We performed a case-control GWAS to identify loci associated with increased risk of small-vessel ischemic stroke in the Han Chinese population using an Affymetrix Axiom CHB array containing 642,832 SNP probes. We initially enrolled 342 SVO stroke and 1,731 controls from a Han Chinese population residing in Taiwan. After kinship analysis and strict quality control filtering, we analyzed 552,090 SNPs (representing 87.9% of array SNPs) for the samples from the GWAS group. Multidimensional scaling analysis (Fig. 1) and results of permutation tests for identity-by-state revealed no differences between the SVO and control groups, providing no evidence for strong population stratification. Quantile–quantile (Q–Q) plots were used to examine *P* value distributions (Fig. 1), and the lambda value was 1.09. In total, we found three top validated SNPs associated with SVO (*P* < 1 × 10^−4^) (Supplementary Table 1). The IBS sharing method implemented in PLINK showed no cryptic family relationships among SVO stroke cases and controls.

### GWAS and cross-platform validation

**Figure 2:**
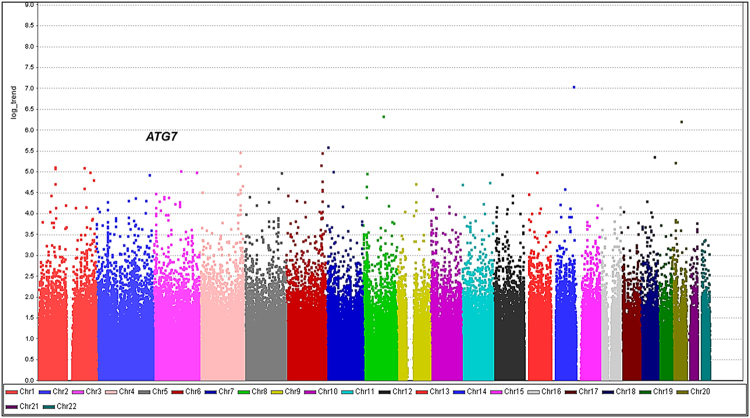
Results of genome-wide association analysis (−log_10_ *P*) shown in chromosomal order for 552,090 SNPs tested for association in initial samples from 342 patients with SVO stroke and 1,731 controls. X-axis represents each of the SNPs used in the primary scan. Y-axis represents the −log_10_ *P* value of the trend test. Signals in the *ATG7* loci are indicated.

The analysis was first performed with samples from 342 individuals with SVO stroke and 1,731 controls (Fig. 2 and Table 2). Qualified Affymetrix calling (>99%) of clustering in both SVO stroke cases and controls, located in (or within) 50 kb of known genes, was selected for cross-platform validation using a Sequenom MassARRAY or direct sequencing.

**Table 2:** SNPs with *P* values < 1 × 10^−5^ in the Joint Analysis.

Chr.	SNP	Position	Gene	Allele format	Risk allele	Stage	control/case	RAF controls	RAF cases	Trend *P*	OR	95%CI	
3	rs2594966	11325276	*ATG7*	GA		GWAS	1731/342	0.6155	0.6959	7.00E-05	1.43	1.198	1.706
				GA	A	Replication	1265/188	0.6105	0.6765	1.31E-02	1.334	1.059	1.681
				GA		Combined	2996/530	0.6133	0.689	2.52E-06	1.397	1.214	1.607
3	rs2594973	11395821	*ATG7*	CG		GWAS	1731/342	0.5948	0.6795	3.24E-05	1.445	1.212	1.722
				CG	G	Replication	1265/188	0.5918	0.6489	3.38E-02	1.275	1.017	1.598
				CG		Combined	2996/530	0.5935	0.6686	3.81E-06	1.381	1.203	1.587
3	rs4684776	11443223	*ATG7*	CT		GWAS	1731/342	0.6096	0.6877	1.08E-04	1.41	1.183	1.681
				CT	T	Replication	1265/188	0.6067	0.6729	1.19E-02	1.334	1.06	1.678
				CT		Combined	2996/530	0.6084	0.6824	3.59E-06	1.383	1.203	1.59
3	rs4686799	186451236	*KNG1*	TC		GWAS	1731/342	0.6598	0.7108	9.95E-03	1.268	1.057	1.521
				TC	C	Replication	1265/188	0.6454	0.7486	1.06E-04	1.637	1.276	2.099
				TC		Combined	2996/530	0.6537	0.7244	8.91E-06	1.392	1.202	1.612
4	rs78868369	126077143	*ANKRD50, FAT4*	TC		GWAS	1731/342	0.08449	0.1268	4.81E-04	1.574	1.219	2.033
				TC	T	Replication	1265/188	0.08796	0.133	4.55E-03	1.59	1.146	2.208
				TC		Combined	2996/530	0.08595	0.129	7.61E-06	1.575	1.288	1.927
6	rs536348	168098215	LOC441178, C6orf123	TC		GWAS	1731/342	0.6407	0.7132	2.52E-04	1.395	1.165	1.67
				TC	C	Replication	1265/188	0.6395	0.7234	1.26E-03	1.474	1.16	1.874
				TC		Combined	2996/530	0.6402	0.7169	1.12E-06	1.423	1.232	1.643
8	rs17201317	104096256	*ATP6V1C1, BAALCOS*	CT		GWAS	1731/342	0.1096	0.1485	3.45E-03	1.417	1.118	1.796
				CT	C	Replication	1265/188	0.1093	0.1738	2.99E-04	1.715	1.276	2.304
				CT		Combined	2996/530	0.1095	0.1575	6.67E-06	1.521	1.265	1.829
14	rs11846182	80872021	*DIO2-AS1*	CT		GWAS	1731/342	0.602	0.6754	2.58E-04	1.376	1.156	1.638
				CT	T	Replication	1265/188	0.6135	0.6818	9.98E-03	1.35	1.07	1.702
				CT		Combined	2996/530	0.6069	0.6777	9.48E-06	1.362	1.185	1.565

Chr., chromosome; gene, genes containing the SNP or the closest gene up to 50 kb upstream or downstream of the SNP; RAF controls, risk allele frequency in controls; RAF cases, risk allele frequency in SVO stroke; OR, odds ratio; 95% CI, 95% confidence interval.

### Replication of top variants for SVO

In the replication stage, 33 SNPs (Supplementary Table 1) were replicated in an independent cohort of 188 patients with SVO stroke and 1,265 controls (Supplementary Table 2). In a combined analysis of the GWAS and replication cohorts, *P* values for 8 of the identified SNPs were lower than 10^−5^ (Table 2).

We found that the SNPs rs2594966 (*P* = 2.52 × 10^−6^), rs2594973 (*P* = 2.52 × 10^−6^), and rs4684776 (*P* = 2.52 × 10^−6^) located at 3p25.3 in *ATG7* (encoding Autophagy Related 7). Other 5 SNPs were located at 3q27.3 (rs4686799, *P* = 8.9 × 10^−6^) in *KNG1* (encoding Kininogen-1), at 4q28.1 (rs78868369, *P* = 7.6 × 10^−6^) in *ANKRD50* (encoding Ankyrin repeat domain-containing protein 50), at 6q27 (rs536348, *P* = 1.1 × 10^−6^) in *LOC441178*, at 8q22.3 (rs17201317, *P* = 6.7 × 10^−6^) in *ATP6V1C1* (encoding ATPase H + transporting V1 subunit C1), and at 14q31.1 (rs11846182, *P* = 9.5 × 10^−6^) in *DIO2-AS1* (encoding DIO2 antisense RNA 1), respectively. These were all replicated in the independent Han Chinese population (Table 1).

**Figure 3:**
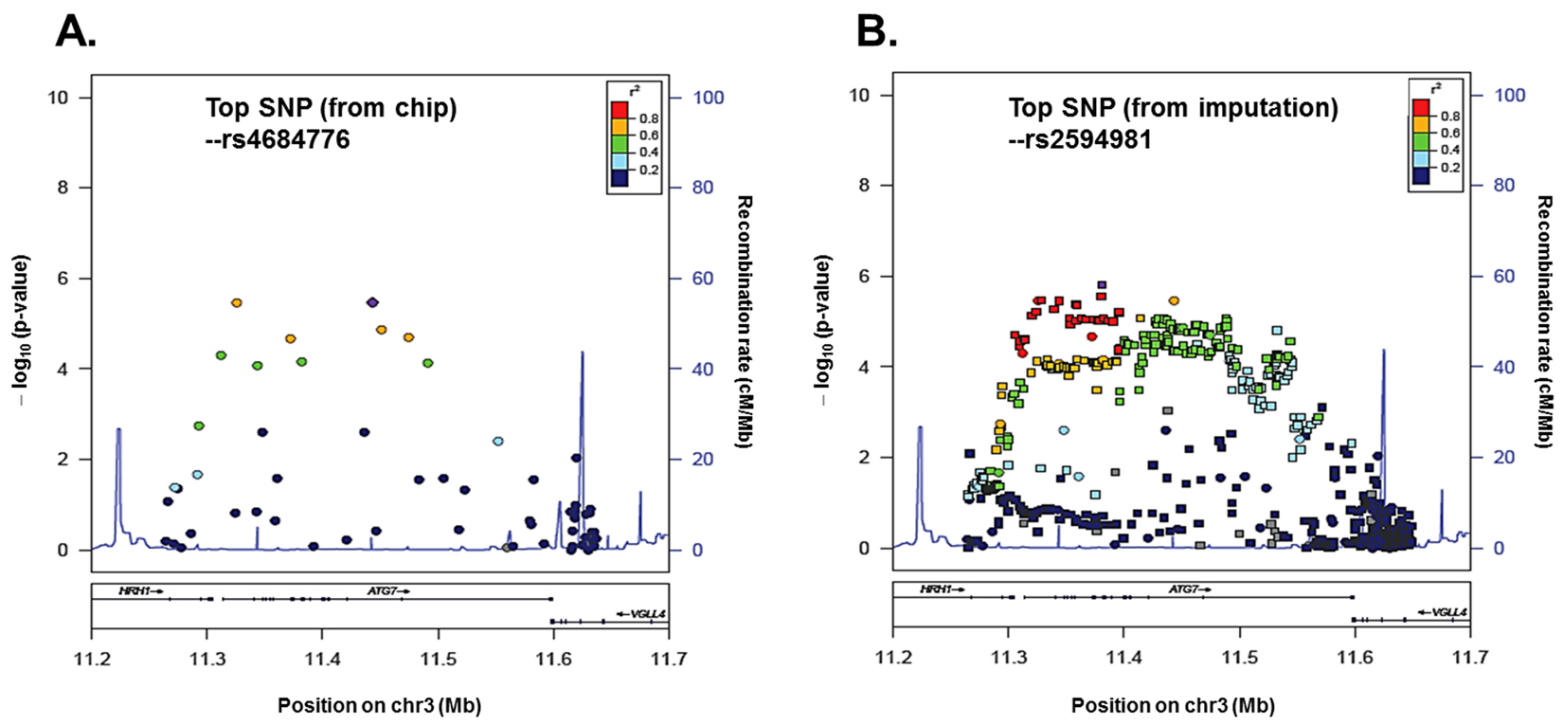
Association plots for the *ATG7* locus. Regional association plot for the *ATG7* locus on chromosome 3 (**A**) with gene annotations superimposed. Each SNP is plotted with respect to its chromosomal location (x-axis) and its −log_10_ *P* values (left y-axis) for the trend test from the primary GWAS scan at that region of the chromosome. After imputation (**B**), squares represent imputed SNPs, and circles represent genotyped SNPs. Colors denote the strength of the linkage disequilibrium of the SNPs to *ATG7*.

In addition, to enhance the SNP coverage, whole gene region of ATG7 was identified using discovery GWAS dataset (Fig. 3). The imputation demonstrated a strong association within one LD with identified the top SNPs including rs2594966, rs2594973, and rs4684776. The top imputed SNP, rs2594981, was further validated by direct genotyping in discovery GWAS. These data also supported the association between ATG7 and SVO stroke.

## Discussion

In this study, we identified novel genetic variants associated with SVO stroke susceptibility. This represents the first report of a GWAS for SVO stroke conducted on a Han Chinese population. Based on two independent Han Chinese groups and without significant difference in other risk factors, several novel loci for SVO stroke were identified and replicated. These findings suggest that SVO is a heritable trait and provide new insights into the genetic basis of SVO stroke.

Although there have been studies showing that autophagy may be involved in stroke^19,20^, the genetic association between autophagy-related genes and SVO stroke was never reported. In the present study, we identified five SVO stroke-associated SNPs—rs2594966, rs2594973, rs4684776, rs34843621, and rs12637318—in the same linkage disequilibrium (LD) of chromosome region 3p25.3. The SNPs are located in the *ATG7* gene (Supplementary Fig. 1), which encodes an ubiquitin-activating E1-like enzyme critical for autophagy^21^. This newly identified genetic link may reveal novel molecular insights into the pathogenesis of SVO stroke. A recent study revealed that deletion of *ATG7* was strongly protective against neuronal damage in the brain^22^. A megakaryocyte- and platelet-specific deletion of *ATG7* caused modest defects in platelet aggregation and granule cargo packaging in a mouse model^23^, suggesting that *ATG7* may be a contributing factor for thrombosis. Moreover, *ATG7*-dependent autophagy has been related to hepatic lipid metabolism^24^, which has in turn been found to be associated with stroke^25,26^. It is therefore possible that enhancement of SVO stroke by alteration of the lipid profile may be mediated by *ATG7*-dependent autophagy. The potential role of *ATG7* in occlusion and SVO stroke pathogenesis will require further investigation. Another SVO-associated SNP, rs4686799, was identified within an intron of the *KNG1* gene. *KNG1* was identified from a GWAS for plasma factor XI levels as a genetic determinant of activated partial thromboplastin time^27^. Elevated plasma levels of FXI have been correlated with venous thrombosis and ischemic stroke; therefore, *KNG1* could be involved in the pathogenesis of SVO stroke via regulation of plasma FXI levels.

A meta-analysis showed that SNP 1425 G/A in *PRKCH* was associated with ischemic stroke, particularly lacunar infarction, in Chinese and Japanese populations^28^ and *ALDH2* ^29,30^ and *FOXF2* ^29,30^ with small vessel disease and white matter hyperintensity in Caucasians, respectively. The disparity in risk genes between Asian and Caucasian populations could be due to the inconsistency in diagnostic and stroke subtyping criteria among centers and nations. The current study was conducted in a single healthcare system using similar diagnostic tools, and the SVO subtype was classified by single doctor.

A major limitation of the current study could be the sample size. However, because SVO was reported to be common in patients with intracranial artery stenosis^31^, the current study used strict recruitment criteria to exclude cases with extracranial and/or intracranial artery stenosis; hence, the sample size was reduced. An additional independent larger group, such as the International Stroke Genetics Consortium, CHARGE, or METASTROKE, would strengthen our findings with detailed stroke subtyping. Further genome-wide association tests assessing whether the potential susceptibility loci have genome-wide significance in different populations will elucidate the genetic contribution in SVO stroke pathogenesis.

In this study, we provide the first genome-wide evidence showing in two independent cohorts, thirty-three SNPs located in novel genetic loci that were found to be associated with SVO stroke in a Han Chinese population. The novel risk loci for SVO stroke contained genes, especially for *ATG7*, that have been implicated in autophagy and thrombosis, which may provide insights into future studies to identify the therapeutic targets for SVO stroke.

## Electronic supplementary material

Supplementary information
