# Supplementary material for: A genome-wide association study links small-vessel ischemic stroke to autophagy
Source: Sci Rep. 2017 Nov 9;7:15229. doi: 10.1038/s41598-017-14355-3 (PMC5680343; doi:10.1038/s41598-017-14355-3)
Supplement: Supplementary file 1 — Supplementary information [file 41598_2017_14355_MOESM1_ESM.pdf]

A genome-wide association study links small-vessel ischemic stroke to autophagy

*Tsong-Hai Lee[1], MD, PhD; *Tai-Ming Ko[2,3], PhD; Chien-Hsiun Chen[2,4], PhD; Yeu-Jhy Chang[1], MD; Liang-Suei Lu[2], MS; Chien-Hung Chang[1], MD; Kuo-Lun Huang[1], MD; Ting-Yu Chang[1], MD; Jiann-Der Lee[5], MD; Ku-Chou Chang[6], MD; Jen-Tsung Yang[5], MD, PhD; Ming-Shien Wen[1], MD; Chao-Yung Wang[1], MD; Ying-Ting Chen[2], MS; Tsai-Chuan Chen[2], MS; Shu-Yu Chou[1], MS; Ming-Ta Michael Lee[7], PhD; Yuan-Tsong Chen[2,8], MD, PhD; Jer-Yuarn Wu[2,4], PhD

[1] Chang Gung Memorial Hospital, Linkou Medical Center, and Chang Gung University College of Medicine, Taoyuan, Taiwan.

[2] Institute of Biomedical Sciences, Academia Sinica, Taipei, Taiwan.

[3] Graduate Institute of Integrated Medicine, College of Chinese Medicine, China Medical University, Taichung, Taiwan.

[4] School of Chinese Medicine, China Medical University, Taichung, Taiwan.

[5] Chang Gung Memorial Hospital, Chiayi Branch, and Chang Gung University College of Medicine, Chiayi, Taiwan.

[6] Chang Gung Memorial Hospital, Kaohsiung Medical Center, and Chang Gung University College of Medicine, Kaohsiung, Taiwan.

[7] Genomic Medicine Institute, Geisinger Health System, Danville, Pennsylvania, USA.

[8] Department of Pediatrics, Duke University Medical Center, Durham, North Carolina, USA.

These authors contributed equally to this work: Tsong-Hai Lee, Tai-Ming Ko

Correspondence should be addressed to:

Yuan-Tsong Chen, MD., PhD. Institute of Biomedical Sciences, Academia Sinica, 128, Academia Road, Section 2. Nankang, Taipei 11529, Taiwan; e-mail: chen0010@ibms.sinica.edu.tw ; Phone: +886-2-27899081; Fax: +886-2-27899085

Jer-Yuarn Wu, PhD. Institute of Biomedical Sciences, Academia Sinica, 128, Academia Road, Section 2. Nankang, Taipei 11529, Taiwan; e-mail: jywu@ibms.sinica.edu.tw ; Phone: +886-2-27899075

Supplementary Table 1. Summary of discovery studies of top 33 SNPs in trend tests.

Chr.	Position	SNP	Gene	Allele 1	Allele 2	Risk allele	RAF controls	RAF cases	Discovery Trend *P*	Risk allele OR	95%CI	
2	742685	rs60477084	*TMEM18, LINC01115*	A	G	A	0.3326	0.4065	1.70E-04	1.374	1.16	1.628
2	57922033	rs6732639	*VRK2, CCDC85A*	C	T	C	0.2182	0.2794	4.35E-04	1.39	1.154	1.674
2	120187344	rs11123523	*TMEM37, DBI*	A	G	A	0.4236	0.4868	2.18E-03	1.291	1.095	1.522
2	120191460	rs2579631	*TMEM37*	C	T	C	0.4222	0.4839	2.91E-03	1.283	1.089	1.513
2	129505509	rs3907871	*HS6ST1, LOC101927881*	T	C	C	0.6952	0.7559	1.33E-03	1.358	1.123	1.642
2	146779043	rs12479100	*PABPC1P2, TEX41*	C	T	C	0.104	0.153	2.27E-04	1.557	1.227	1.977
3	11325276	rs2594966	*ATG7*	G	A	A	0.6155	0.6959	7.00E-05	1.43	1.198	1.706
3	11395821	rs2594973	*ATG7*	C	G	G	0.5948	0.6795	3.24E-05	1.445	1.212	1.722
3	11443223	rs4684776	*ATG7*	C	T	T	0.6096	0.6877	1.08E-04	1.41	1.183	1.681
3	11450873	rs34843621	*ATG7*	G	A	A	0.6914	0.7625	1.79E-04	1.433	1.184	1.734
3	11474823	rs12637318	*ATG7*	T	C	C	0.693	0.7625	2.50E-04	1.422	1.176	1.721
3	140492139	rs3923751	*SLC25A36, TRIM42*	G	A	A	0.9094	0.9515	2.65E-04	1.952	1.351	2.823
3	140527761	rs74282906	*SLC25A36, TRIM42*	G	T	T	0.9097	0.9518	2.62E-04	1.958	1.354	2.83
3	186451236	rs4686799	*KNG1*	T	C	C	0.6598	0.7108	9.95E-03	1.268	1.057	1.521
4	126077143	rs78868369	*ANKRD50, FAT4*	T	C	T	0.08449	0.1268	4.81E-04	1.574	1.219	2.033
4	175913798	rs10026079	*ADAM29, GPM6A*	T	C	C	0.9286	0.9575	6.28E-03	1.731	1.168	2.566
4	175919987	rs10005382	*ADAM29, GPM6A*	G	C	C	0.9283	0.9576	5.51E-03	1.745	1.177	2.587
5	81778496	rs224844	*MIR3977, ATP6AP1L*	T	C	T	0.455	0.5297	4.63E-04	1.349	1.143	1.592
5	139539514	rs2291878		G	A	G	0.1969	0.2574	2.91E-04	1.413	1.167	1.711
6	168098215	rs536348	*LOC441178, C6orf123*	T	C	C	0.6407	0.7132	2.52E-04	1.395	1.165	1.67
8	6067096	rs73188691	*CSMD1, LOC100287015*	C	A	C	0.07093	0.1033	2.72E-03	1.51	1.138	2.003
8	104096256	rs17201317	*ATP6V1C1, BAALCOS*	C	T	C	0.1096	0.1485	3.45E-03	1.417	1.118	1.796
10	70380498	rs12764361	*TET1*	A	C	A	0.1288	0.1672	7.05E-03	1.358	1.085	1.7
10	72944439	rs117743525	*PCBD1, UNC5B*	T	G	G	0.9324	0.9603	6.60E-03	1.753	1.167	2.634
11	100057536	rs11223142	*CNTN5*	G	A	G	0.4477	0.5294	9.72E-05	1.388	1.177	1.637
12	12810328	rs2900298	*CREBL2, GPR19*	C	G	G	0.4991	0.5718	6.37E-04	1.34	1.136	1.582
12	15176510	rs2263590	*LOC101928340, RERG*	A	G	A	0.2693	0.3413	1.93E-04	1.406	1.178	1.678
12	58097750	rs75013973	*OS9*	G	A	G	0.141	0.1969	2.60E-04	1.494	1.204	1.853
13	59812089	rs9570072	*LOC101926897, DIAPH3*	C	T	T	0.8327	0.8864	4.56E-04	1.569	1.217	2.021
14	22587753	rs2141974	*OR4E2, DAD1*	C	T	C	0.07259	0.1147	2.65E-04	1.655	1.26	2.174
14	80872021	rs11846182	*DIO2-AS1*	C	T	T	0.602	0.6754	2.58E-04	1.376	1.156	1.638
15	82234966	rs7163891	*LOC101929655, MEX3B*	C	T	C	0.07423	0.1147	4.28E-04	1.616	1.236	2.112
19	575964	rs2238542	*BSG*	T	C	T	0.2188	0.2657	8.07E-03	1.291	1.069	1.561

Supplementary Table 2. Summary of replication studies of top 33 SNPs in trend tests.

Chr.	Position	SNP	Gene	Allele 1	Allele 2	Risk allele	RAF controls	RAF cases	Discovery Trend *P*	Risk allele OR	95%CI	
2	742685	rs60477084	*TMEM18, LINC01115*	A	G	A	0.347	0.4016	4.08E-02	1.263	1.011	1.577
2	57922033	rs6732639	*VRK2, CCDC85A*	C	T	C	0.2146	0.266	2.51E-02	1.326	1.035	1.699
2	120187344	rs11123523	*TMEM37, DBI*	A	G	A	0.4243	0.4973	7.79E-03	1.342	1.08	1.668
2	120191460	rs2579631	*TMEM37*	C	T	C	0.4202	0.4973	5.03E-03	1.365	1.098	1.697
2	129505509	rs3907871	*HS6ST1, LOC101927881*	T	C	C	0.6979	0.7633	9.60E-03	1.396	1.084	1.797
2	146779043	rs12479100	*PABPC1P2, TEX41*	C	T	C	0.0949	0.129	4.06E-02	1.413	1.015	1.968
3	11325276	rs2594966	*ATG7*	G	A	A	0.6105	0.6765	1.31E-02	1.334	1.059	1.681
3	11395821	rs2594973	*ATG7*	C	G	G	0.5918	0.6489	3.38E-02	1.275	1.017	1.598
3	11443223	rs4684776	*ATG7*	C	T	T	0.6067	0.6729	1.19E-02	1.334	1.06	1.678
3	11450873	rs34843621	*ATG7*	G	A	A	0.6839	0.738	3.35E-02	1.302	1.019	1.664
3	11474823	rs12637318	*ATG7*	T	C	C	0.6865	0.742	2.87E-02	1.314	1.027	1.68
3	140492139	rs3923751	*SLC25A36, TRIM42*	G	A	A	0.9233	0.9521	4.75E-02	1.652	1.006	2.711
3	140527761	rs74282906	*SLC25A36, TRIM42*	G	T	T	0.9233	0.9521	4.72E-02	1.653	1.007	2.714
3	186451236	rs4686799	*KNG1*	T	C	C	0.6454	0.7486	1.06E-04	1.637	1.276	2.099
4	126077143	rs78868369	*ANKRD50, FAT4*	T	C	T	0.08796	0.133	4.55E-03	1.59	1.146	2.208
4	175913798	rs10026079	*ADAM29, GPM6A*	T	C	C	0.9229	0.9681	1.71E-03	2.535	1.401	4.589
4	175919987	rs10005382	ADAM29, GPM6A	G	C	C	0.9225	0.9654	3.01E-03	2.345	1.323	4.155
5	81778496	rs224844	*MIR3977, ATP6AP1L*	T	C	T	0.4419	0.5133	1.05E-02	1.332	1.072	1.655
5	139539514	rs2291878		G	A	G	0.1883	0.242	1.45E-02	1.376	1.065	1.778
6	168098215	rs536348	LOC441178, C6orf123	T	C	C	0.6395	0.7234	1.26E-03	1.474	1.16	1.874
8	6067096	rs73188691	CSMD1, LOC100287015	C	A	C	0.06926	0.1117	3.05E-03	1.69	1.184	2.413
8	104096256	rs17201317	*ATP6V1C1, BAALCOS*	C	T	C	0.1093	0.1738	2.99E-04	1.715	1.276	2.304
10	70380498	rs12764361	*TET1*	A	C	A	0.1296	0.1962	5.56E-04	1.639	1.238	2.171
10	72944439	rs117743525	*PCBD1, UNC5B*	T	G	G	0.9324	0.9814	1.75E-04	3.824	1.782	8.208
11	100057536	rs11223142	*CNTN5*	G	A	G	0.453	0.5134	2.80E-02	1.274	1.025	1.583
12	12810328	rs2900298	*CREBL2, GPR19*	C	G	G	0.5028	0.5695	1.71E-02	1.308	1.051	1.629
12	15176510	rs2263590	*LOC101928340, RERG*	A	G	A	0.2882	0.3431	2.91E-02	1.29	1.025	1.623
12	58097750	rs75013973	*OS9*	G	A	G	0.1308	0.1685	4.68E-02	1.346	0.9998	1.812
13	59812089	rs9570072	*LOC101926897, DIAPH3*	C	T	T	0.827	0.8803	9.36E-03	1.539	1.108	2.136
14	22587753	rs2141974	*OR4E2, DAD1*	C	T	C	0.06365	0.09358	2.73E-02	1.519	1.035	2.228
14	80872021	rs11846182	*DIO2-AS1*	C	T	T	0.6135	0.6818	9.98E-03	1.35	1.07	1.702
15	82234966	rs7163891	*LOC101929655, MEX3B*	C	T	C	0.06759	0.09893	2.71E-02	1.515	1.043	2.2
19	575964	rs2238542	*BSG*	T	C	T	0.2233	0.3059	4.53E-04	1.533	1.207	1.946

Supplementary Figure 1:

A.

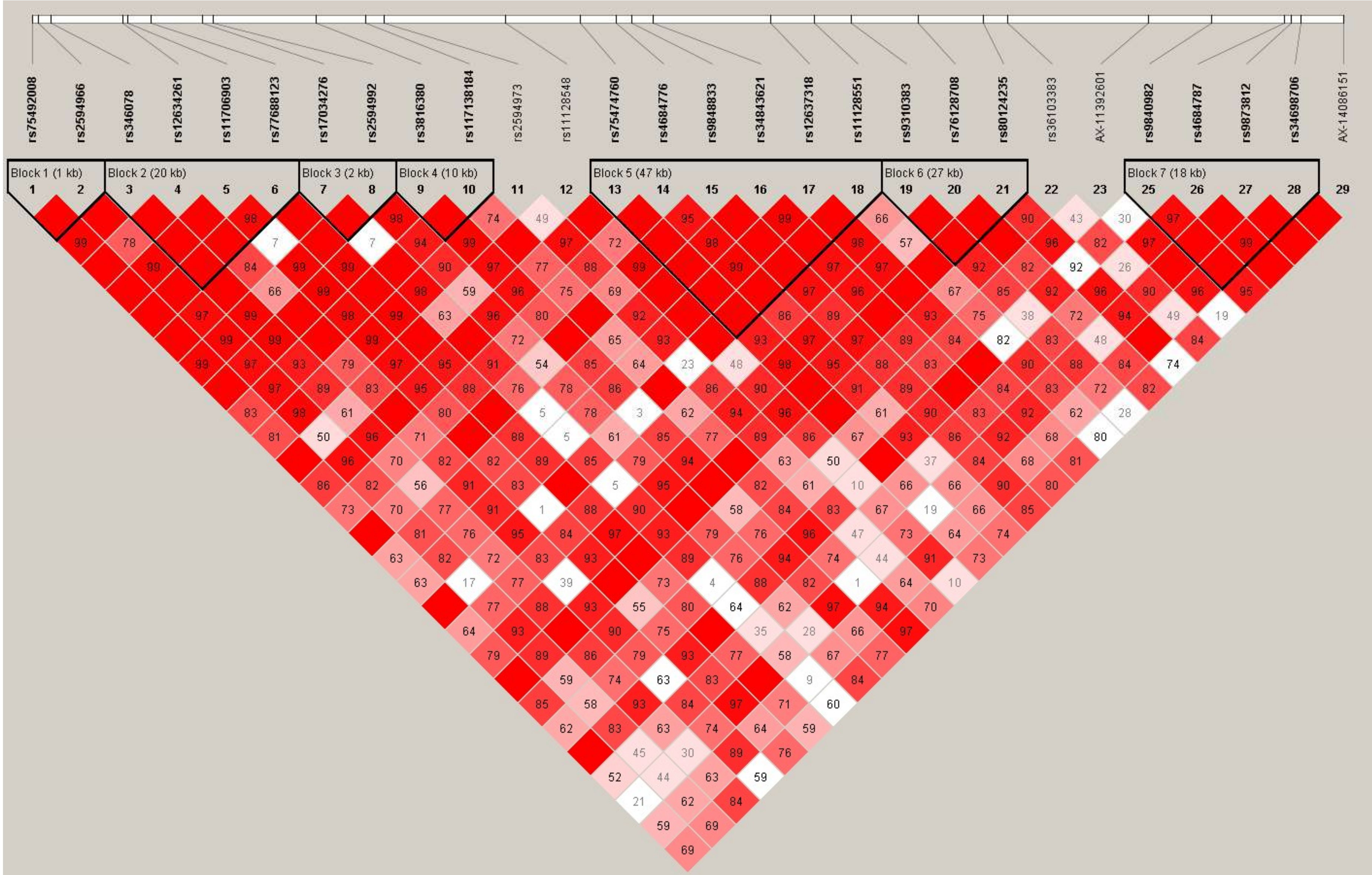

Supplementary Figure 1:

B.

ATG7 SNP1	ATG7 SNP2	D'	r^2
rs2594966	rs2594973	0.987	0.893
rs2594966	rs4684776	0.820	0.655
rs2594966	rs34843621	0.818	0.48
rs2594966	rs12637318	0.827	0.486
rs2594973	rs4684776	0.881	0.729
rs2594973	rs34843621	0.924	0.564
rs2594973	rs12637318	0.937	0.573
rs4684776	rs34843621	0.983	0.678
rs4684776	rs12637318	0.993	0.683
rs34843621	rs12637318	0.998	0.984

Supplementary Figure 1: LD structure and logistic regression analyses within the *ATG7* region. (A) –log10 (*P* values) of SNPs for the best test from the primary scan as a function of genomic positions for the *ATG7* region. (B) LD patterns, D’ value and r^2 among the disease-associated SNPs within the *ATG7* region (Chr3: 11,313,995-11,599,139 based on ensemble version v.GRCh37).
